# Insights into the lemon (*Citrus limon*) epiphytic microbiome: impact of the biocontrol yeast *Clavispora lusitaniae* 146

**DOI:** 10.1186/s13104-024-07064-4

**Published:** 2025-01-13

**Authors:** Maria Cecilia Rasuk, José Matías Irazoqui, María Florencia Perez, Martina María Pereyra, Pedro Eugenio Sineli, Anja Poehlein, Rolf Daniel, Julian Rafael Dib

**Affiliations:** 1 Planta Piloto de Procesos Industriales Microbiológicos (PROIMI – CONICET), Tucumán, Argentina; 2 Instituto de Investigación de la Cadena Láctea (IDICAL, INTA-CONICET), Rafaela, 2300 Argentina; 3 Genomic and Applied Microbiology & Göttingen Genomics Laboratory, Institute of Microbiology and Genetics, Georg-August University of Göttingen, 37077 Göttingen, Germany; 4 Instituto de Microbiología, Facultad de Bioquímica, Química y Farmacia, Universidad Nacional de Tucumán (UNT), Tucumán, Argentina

**Keywords:** Microbiome, Lemon, Biocontrol, Postharvest, *Clavispora*

## Abstract

**Background:**

Postharvest lemons are affected by several fungal infections, and as alternatives to chemical fungicides for combating these infections, different microbial biocontrol agents have been studied, with the *Clavispora lusitaniae* 146 strain standing out. Although strain 146 has proven to be an effective agent, the influence of a microbial biological control agent on the postharvest lemon microbiome has not been studied until now. Thus, this study aimed to evaluate how the epiphytic microbiome of postharvest lemons is affected by the application of the biocontrol yeast *C. lusitaniae* 146.

**Results:**

In terms of bacterial composition, the most abundant genera were *Sphingomonas*, *Pelomonas*, and *Bacillus* and no significant differences in the composition were detected between the treated and control samples. Among fungi, *Clavispora* was predominant not only in the treated samples but also in the control, and statistics indicated differences, suggesting its significant role in modulating the epiphytic community composition of lemon. Understanding fruit microbiomes is vital for effective disease control, and this study provides insights into the microbial composition of the surface of lemon and the role of *C. lusitaniae* 146.

**Supplementary Information:**

The online version contains supplementary material available at 10.1186/s13104-024-07064-4.

## Introduction

Lemons (*Citrus limon*) are among the most important perennial fruit crops in the world. They are usually affected by various phytopathogenic fungi, including species from the genus *Penicillium*, the causal agents of green and blue mold, and species from *Alternaria*, the causal agents of black rot [1–3]. To combat fungal decay, synthetic chemical fungicides are commonly used. However, their extensive application has led to resistance among phytopathogenic fungal strains [4] and has raised concerns regarding human health and environmental impacts. In pursuit of more sustainable and environmentally friendly agriculture, the study of the microbiome has started to play a significant role [5]. Thus, understanding the composition and function of these microorganisms is essential for promoting plant health, enhancing crop productivity, and reducing reliance on chemical inputs such as pesticides and fertilizers [6]. Previous studies have characterized the structural and functional diversity of rhizosphere microbial communities from citrus plants and evaluated how a phytopathogen influences these communities [7–9]. Despite these advances, research on the microbiome of citrus fruits remains limited, underscoring the need for further investigation into microbial dynamics during the postharvest stage. Gomba et al. (2017) explored the effects of commercial citrus packhouse processing steps on the fruit surface microbiome of oranges (*Citrus sinensis*), identifying key bacterial and fungal taxa that influence fruit quality and shelf-life [10]. Similarly, Kumar et al. (2021) investigated the postharvest microbiome of citrus fruits (*Citrus reticulata*), focusing on the dynamics of microbial populations during storage and their impact on fruit decay [11]. More recently, Jing et al. (2023) examined the microbiome of four citrus fruit varieties (*Citrus unshiu*, *Citrus reticulata*, *Citrus sinensis* and *Citrus limon*) to design a microbial community with biocontrol effects against citrus postharvest disease by mixing selected strains [12]. While biological control agents like the yeast *Clavispora lusitaniae* 146 have shown the ability to control postharvest fungal pathogens on lemons [13–15], our understanding of the lemon microbiome and how it is influenced by biocontrol agents remains limited. Thus, this study aims to characterize the lemon epiphytic microbiome and investigating the impact of *C. lusitaniae* 146 on its composition and diversity. By employing a culture-independent metagenomic approach, analyzing both the 16S rRNA gene and the ITS region, we provide novel insights into the complex microbial ecosystem of lemons and potential insights into the underlying biocontrol efficacy.

## Methodology

### Experimental design

Eight lemons of the Eureka cultivar (*Citrus limon* (L.) Burm) were harvested from a local field in La Cocha, Tucumán, Argentina (27°46′16″S − 65°35′08″O). Each lemon was devoid of any visible injury or signs of decay and exhibited uniformity in size, shape, and ripeness (green-yellow stage). Four lemons were subjected to treatment with *C. lusitaniae* 146 by immersion (T) as described previously [16]. The remaining four lemons served as untreated controls (C). All fruits were stored at 25 °C for 7 days, after which a pool of three pieces of peel (1 cm^2^) was extracted with a scalpel from each lemon for DNA extraction. To maintain sterility, all equipment was washed and surface sterilized with 70% ethanol between samples. All the samples were stored frozen until further use.

### DNA extraction

Total DNA was extracted from the lemon samples via the MasterPure™ Complete DNA and RNA Purification Kit according to the manufacturer’s instructions (Epicenter, Wisconsin, USA).

### Sequencing

PCR amplification, library construction, and sequencing were carried out by the Göttingen Genomics Laboratory at Georg August University of Göttingen, Germany. Bacterial 16S rRNA gene amplicons were generated using primers targeting the V3–V4 region [17], while fungal ITS amplicons were obtained using primers for the ITS2 region [18]. Sequencing was performed using the Illumina MiSeq platform.

### Bioinformatic analysis

Both datasets (Fungi and Bacteria) were processed separately. The DADA2 algorithm [19] was executed in QIIME2 [20] to quality filter reads and remove chimeric sequences. The resulting amplicon sequence variants (ASVs) were classified via the naive Bayesian classifier provided by QIIME2 and compared against the SILVA database (v138.1) and UNITE database (v9.0) [21–23]. Alpha diversity indices (observed features, Faith and Shannon) and beta diversity indices (unweighted and weighted UniFrac) were calculated via QIIME2. Principal coordinate analysis (PCoA) plots based on UniFrac distance metrics were generated for the two populations. To assess differences in both alpha and beta diversity between treatments, Kruskal‒Wallis and PERMANOVA tests were used, respectively. Finally, the differences in taxon abundance between both groups of samples (control and treated) were statistically evaluated via the Kruskal‒Wallis test.

## Results

### Characteristics of the sequences

A total of 297,827 reads were obtained for the 16S rRNA gene amplicons, and 277,523 were obtained for the ITS region (Supplementary Table S1). After paired-end alignment, quality filtering, and deletion of chimeric and nontarget sequences, 82 different bacterial ASVs were identified. In contrast, a much greater number of ASVs (2589) were obtained for fungi. The rarefaction curves and saturation of these curves indicated that the survey size for all the samples was sufficient to assess the overall bacterial and fungal diversity and community composition.

### Effect of treatment with *C. Lusitaniae* 146 on the lemon microbiome

**Fig. 1:**
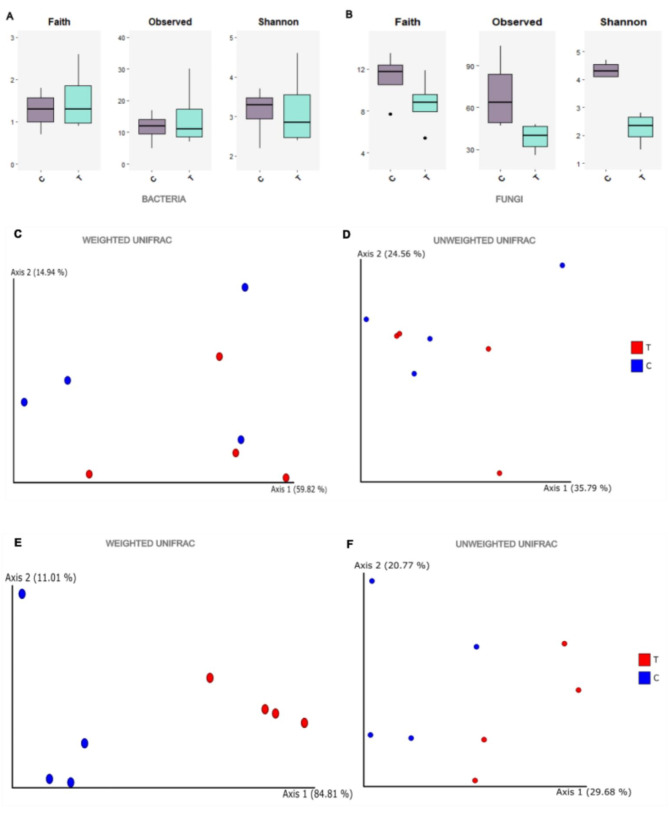
Box plots of the bacterial (**A**) and fungal (**B**) diversity (Faith, observed features and Shannon indices) in the lemon samples. C: control; T: treatment with yeast 146. Principal coordinate analysis (PCoA) of bacterial (**C - D**) and fungal (**E - F**) populations associated with lemon samples on the basis of UniFrac beta diversity distance metrics. Analyses were performed considering sequences from untreated control samples (blue) and samples treated with yeast 146 (red)

Alpha diversity indices (observed features, Faith´s phylogenetic diversity and Shannon index) were utilized to evaluate the alpha diversity of bacterial and fungal communities within the samples. Bacterial observed features ranged from 5 to 30 in both the treated and control samples. Shannon diversity remained unaffected by the application of *C. lusitaniae* 146, as evidenced by the Kruskal‒Wallis test results (*P* = 1). On the other hand, the fungal population was significantly more diverse in the control samples than in those treated with yeast, according to the Shannon and observed features indices (*P* < 0.05) (Fig. 1, Supplementary Table S2).

To further explore differences in microbial community composition between the two groups of samples, PCoA plots based on UniFrac distance metrics were generated for the two populations (Fig. 1). The results of the PERMANOVA revealed a significant effect of treatment on Fungi (*P* = 0.028), whereas the effect on bacteria was not statistically significant (*P* = 0.66).

**Fig. 2:**
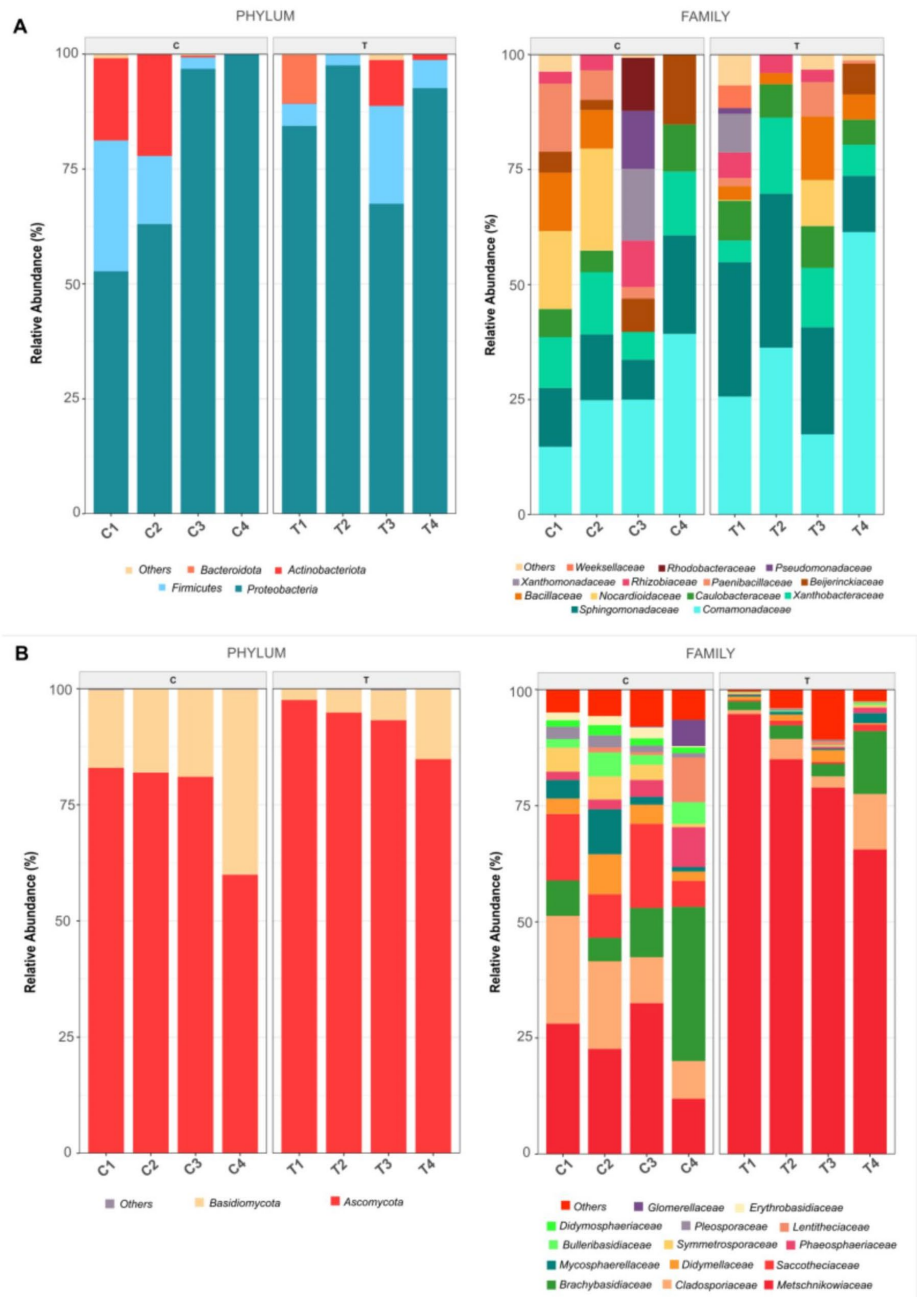
Relative abundances of bacterial (**A**) and fungal (**B**) taxa in lemon samples. C1, C2, C3 and C4 correspond to untreated control samples, and T1, T2, T3 and T4 correspond to samples treated with *C. lusitaniae* 146. Taxa that were less abundant than 1% were included in “Others”

**Taxonomic characterization of the lemon carposphere**: At the phylum level, the bacterial composition was dominated mainly by Proteobacteria (C: 52–100%, T: 67–97%). Additionally, Firmicutes (C: 2–28%, T: 2–21%), Actinobacteria (C: 0–22%, T: 1–10%), and Bacteroidota (C: 0%, T: 20%) were also identified. At the family level, Comamonadaceae (C: 15–39%, T: 17–61%) and Sphingomonadaceae (C: 9–21%, T: 12–33%) were the prevailing taxa. However, other families, such as Xanthobacteraceae, Nocardioidaceae, Caulobacteraceae, Bacillaceae, Paenibacillaceae, Xanthomonadaceae, and Rhizobiaceae, were also present, ranging from 0 to 20% (Fig. 2). Although the effect of the treatment on the bacterial community structure was not statistically significant, the relative abundances of the genera *Sphingomonas* and *Pelomonas* increased, whereas those of *Aquabacterium* and *Nocardioides* decreased (Supplementary Table S3). Other genera also present at high relative abundances included *Bacillus*, *Paenibacillus*, and *Methylorubrum*. Statistical analyses revealed no significant differences between the groups treated with *C. lusitaniae* 146 and the control group, except for *Methylorubrum* (*P* < 0.05). With respect to the fungal community composition, the phyla Ascomycota (C: 60–82%, T: 84–97%) and Basidiomycota (C: 16–39%, T: 2–15%) were dominant. At the family level, Metschnikowiaceae (C: 12–32%, T: 66–95%) was predominant, with *Clavispora* being the main representative genus (Fig. 2). The next most abundant families included Cladosporiaceae (C: 8–23%, T: 1–12%), represented by *Cladosporium*, Brachybasidiaceae (C: 5–33%, T: 2–14%), with *Kordyana* as the only genus, and Saccotheciaceae (C: 6–18%, T: 0–1%), featuring *Aerobasidium* as the sole genus. As expected, the relative abundance of the *Clavispora* genus increased considerably with treatment, reducing that of other genera, such as *Kordyana*, *Cladosporium* and *Aeurobasidium* (Supplementary Table S3). The genera *Alternaria* and *Penicillium* were also detected at low relative abundances, with both decreasing in abundance in the treated samples.

## Discussion

This report describes the epiphytic microbiome of lemons in this region and its variation following treatment with the biocontrol yeast *Clavispora lusitaniae* 146. Our previous studies demonstrated that this yeast strain effectively prevents green mold caused by *Penicillium digitatum*, making it a suitable protective agent for postharvest lemon management [13–16, 24, 25] To our knowledge, this is the first study that describes the response of the epiphytic microbiome of postharvest lemons to a biocontrol agent. The predominant bacterial phyla identified were Proteobacteria, Firmicutes, and Actinobacteria, whereas Ascomycota and Basidiomycota were the main fungal phyla. These results are consistent not only with previous studies on microorganisms isolated from postharvest lemon surfaces, which also identified such phyla [16, 25] but also with these phyla dominating the microbiomes of citrus tree leaves, budwood, root tissues, and oranges [8, 10, 26]. As previously reported, we have repeatedly isolated *Clavispora* strains from local citrus samples that have demonstrated high biocontrol efficacy [15, 27]. This study consistently demonstrated that *Clavispora* is the most abundant genus in the lemon microbiome. These findings underscore the important role of this genus in maintaining lemon health, particularly after harvesting.

The diversity comparison between the treated and control samples revealed that the samples treated with *C*. *lusitaniae* 146 presented lower fungal diversity. Similar results were obtained in cherry tomatoes (*Lycopersicon esculentum*), strawberries (*Fragaria* × *ananassa*), and apples (*Malus domestica*) treated with biocontrol agents [28–30]. However, no significant differences were observed in the bacterial composition between the treated and control samples, which contrasts with findings of other reports, in which significant changes in the bacterial composition of strawberries after field application of the yeast *Metschnikowia fructicola* were detected [31]. The stability of the bacterial microbiome composition and structure suggests that *C. lusitaniae* 146 does not significantly alter the native epiphytic bacterial communities. At the genus level, treatment with *C. lusitaniae* 146 slightly enriched potential beneficial genera such as *Sphingomonas*, *Pelomonas* and *Bacillus*. *Sphingomonas* species are associated with disease suppression and growth promotion during stress conditions [32, 33]. Similarly, *Pelomonas* strains act as multiple stress reducers, bioremediation agents, and growth promoters in essential crops [34]. *Bacillus* species isolated from the rhizosphere and phyllosphere of citrus trees effectively biocontrol citrus-related bacterial diseases [35].

Treatment with *C. lusitaniae* 146 significantly reduced fungal alpha diversity, altering the composition and general structure of the fungal microbiome. In terms of fungal taxonomic composition, at the genus level, *Clavispora* dominated both the treated and control samples. In addition, other abundant genera, including *Kordyana*, *Cladosporium* and *Aerobasidium*, which have antagonistic activities against postharvest pathogens, have been described as promising biocontrol candidates [36–40]. Fungal phytopathogens affecting postharvest lemons were also detected. Although *C*. *lusitaniae* 146 did not significantly reduce the relative abundance of *Penicillium*, it achieved a significant reduction in *Alternaria* abundance. The latter demonstrates a trend towards the suppression of pathogenic microbiota, highlighting the potential of this agent to contribute to postharvest disease management.

## Conclusions

In this study, the bacterial composition was dominated by Proteobacteria, including beneficial genera such as *Sphingomonas*,* Pelomonas* and *Bacillus*. Bacterial composition did not significantly differ between the control and treated samples, and for non-pathogenic fungi indicating that *C. lusitaniae* 146 maintains the native bacterial microbiota. The fungal composition, represented in both the treated and control samples, was mainly dominated by *Clavispora*. Furthermore, the application of yeast 146 promoted the reduction of *Penicillium* and *Alternaria*, two postharvest fungal pathogens. This study provides the first description of the native lemon peel microbiome and insights into the impact of the biocontrol yeast *C. lusitaniae* 146 on this microbiome. The results highlight the potential of *C. lusitaniae* 146 in modulating the fungal but not the bacterial composition of the lemon carposphere microbiome, enhancing our understanding of the effects of a biocontrol agent on the microbiome.

### Limitations

This pilot study has some limitations. The number of sequences derived from chloroplasts and mitochondria was high because of the sampling method, so we designed now a sampling method that avoids excess of host DNA carryover by focusing solely on the lemon surface without taking part in the lemon peel. Additionally, future studies will include incubating lemons at 8 °C to evaluate potential temperature-related differences, and sampling across three different harvest periods would allow a better evaluation of microbiome distribution over time, which is associated with physiological changes in the fruit.

## Electronic supplementary material

Below is the link to the electronic supplementary material.

Supplementary Material 1

## Data Availability

All data is available at GenBank, as part of the bioproject PRJNA1139195. Raw reads for bacterial datasets were deposited in the SRA database as listed next. C1: SRR30000299; C2: SRR30000298; C3: SRR30000297; C4: SRR30000296; T1: SRR30000295; T2: SRR30000294; T3: SRR30000293; T4: SRR30000292. Raw reads for fungal datasets were also deposited in SRA, as listed next. C1: SRR29988896; C2: SRR29988895; C3: SRR29988894; C4: SRR29988893; T1: SRR29988892; T2: SRR29988891; T3: SRR29988890; T4: SRR29988889.
