# Supplementary material for: Insights into the lemon (Citrus limon) epiphytic microbiome: impact of the biocontrol yeast Clavispora lusitaniae 146
Source: BMC Res Notes. 2025 Jan 13;18:11. doi: 10.1186/s13104-024-07064-4 (PMC11730150; doi:10.1186/s13104-024-07064-4)
Supplement: Supplementary file 1 — Supplementary Material 1 [file 13104_2024_7064_MOESM1_ESM.pdf]

SUPPLEMENTARY MATERIAL

"Insights into the lemon epiphytic microbiome: impact of the biocontrol yeast *Clavispora lusitaniae* 146"

Supplementary Table S1. Sequencing and quality filtering results for 16S (bacteria) and ITS (fungi) data. C1, C2, C3 and C4 correspond to untreated control samples; and T1, T2, T3 and T4 to samples treated with yeast 146.

	**BACTERIA**				**FUNGI**			
**Sample_id**	**Reads**	**Denoising**		**Mitoc-Chlo-Filtered**	**Reads**	**Denoising**		**Mitoc-Chlo-Filtered**
**C1**	60778	9616	15.8	915	33729	22157	65.7	2654
**C2**	25531	4999	19.6	406	36480	25222	59.1	2708
**C3**	26944	4754	17.6	564	55209	37602	68.1	13525
**C4**	32168	6942	21.6	224	37137	21001	56.6	8367
**T1**	42332	7236	17.1	1212	27778	21903	78.9	16969
**T2**	32707	6194	18.9	248	27936	20423	73.1	6365
**T3**	50217	8623	17.2	550	25244	19113	75.7	7352
**T4**	27150	4319	15.9	311	34010	22874	67.3	11526

Supplementary Table S2. Alpha diversity of bacterial and fungal communities. C1, C2, C3 and C4 correspond to untreated control samples; and T1, T2, T3 and T4 to samples treated with yeast 146.

	**BACTERIA**			**FUNGI**		
**Sample_id**	**Obs-features**	**Shannon**	**Faith**	**Obs-features**	**Shannon**	**Faith**
**C1**	17	3.7	1.8	47	4.1	11.5
**C2**	11	3.2	1.1	50	4.5	7.7
**C3**	13	3.4	1.5	104	4.7	13.5
**C4**	5	2.2	0.7	77	4.1	12
**T1**	30	4.6	2.6	26	1.5	5.4
**T2**	7	2.4	0.9	34	2.1	8.8
**T3**	13	3.2	1.6	48	2.6	11.9
**T4**	9	2.5	1	46	2.8	8.8

Supplementary Table S3. Relative abundance of genera in lemon peel. C: untreated control; T: treatment with yeast 146.

**Bacteria Genera**	C (%)	T (%)
Sphingomonas (*Alphaproteobacteria*)	8 - 21	12 - 33
Aquabacterium (*Gammaproteobacteria*)	10 - 39	5 - 27
Unclassified (*Alphaproteobacteria*)	6 - 14	5 - 16
Pelomonas (*Gammaproteobacteria*)	0 - 12	3 - 34
Nocardioides (*Actinobacteria*)	0 - 22	0 - 10
Bacillus (*Firmicutes*)	0 - 15	0 - 7
Unclassified (*Alphaproteobacteria*)	0 - 10	2 - 9
Methylobacterium-Methylorubrum (*Alphaproteobacteria*)	2 - 15	0 - 6
**Fungi Genera**		
Clavispora (*Ascomycota*)	12 - 32	66 - 95
Kordyana (*Basidiomycota*)	5 - 33	2 - 14
Cladosporium (*Ascomycota*)	8 - 23	1 - 12
Aeurobasidium (*Ascomycota*)	5 - 18	0 - 1

Datasets: The sequencing data have been deposited in the National Center for Biotechnology Information (NCBI) Sequence Read Archive (SRA) under the BioProject ID PRJNA1139195.
